# Early and Sustained Increases in Leukotriene B_4_ Levels Are Associated with Poor Clinical Outcome in Ischemic Stroke Patients

**DOI:** 10.1007/s13311-019-00787-4

**Published:** 2019-09-13

**Authors:** Su Jing Chan, Mary P. E. Ng, Hui Zhao, Geelyn J. L. Ng, Chuan De Foo, Peter T.-H. Wong, Raymond C. S. Seet

**Affiliations:** 1 Department of Pharmacology, Yong Loo Lin School of Medicine, National University Health System, National University of Singapore, MD3, 16 Medical Drive, Singapore, 117600 Singapore; 2 Department of Medicine, Yong Loo Lin School of Medicine, National University Health System, National University of Singapore, NUHS Tower Block, 1E Kent Ridge Road, Singapore, 119228 Singapore

**Keywords:** Ischemic stroke, leukotriene B_4_, animal models, middle cerebral artery

## Abstract

Leukotriene B_4_ (LTB_4_) has been implicated in ischemic stroke pathology. We examined the prognostic significance of LTB_4_ levels in patients with acute middle cerebral artery (MCA) infarction and their mechanisms in rat stroke models. In ischemic stroke patients with middle cerebral artery infarction, plasma LTB_4_ levels were found to increase rapidly, roughly doubling within 24 h when compared to initial post-stroke levels. Further analyses indicate that poor functional recovery is associated with early and more sustained increase in LTB_4_ rather than the peak levels. Results from studies using a rat embolic stroke model showed increased 5-lipoxygenase (5-LOX) expression in the ipsilateral infarcted cortex compared with sham control or respective contralateral regions at 24 h post-stroke with a concomitant increase in LTB_4_ levels. In addition, neutrophil influx was also observed in the infarcted cortex. Double immunostaining indicated that neutrophils express 5-LOX and leukotriene A_4_ hydrolase (LTA_4_H), highlighting the pivotal contributions of neutrophils as a source of LTB_4_. Importantly, rise in plasma LTB_4_ levels corresponded with an increase in LTB_4_ amount in the infarcted cortex, thereby supporting the use of plasma as a surrogate for brain LTB_4_ levels. Pre-stroke LTB_4_ loading increased brain infarct volume in tMCAO rats. Conversely, administration of the 5-LOX-activating protein (FLAP) inhibitor BAY-X1005 or B-leukotriene receptor (BLTR) antagonist LY255283 decreased the infarct volume by a similar extent. To conclude, targeted interruption of the LTB_4_ pathway might be a viable treatment strategy for acute ischemic stroke.

**Electronic supplementary material:**

The online version of this article (10.1007/s13311-019-00787-4) contains supplementary material, which is available to authorized users.

## Introduction

Leukotrienes (LTs) are short-lived but potent proinflammatory lipid mediators that are expressed in macrophages, neutrophils, and mast cells. LTs are derived from arachidonic acid by the sequential actions of cytosolic phospholipase A_2_ and 5-lipoxygenase (5-LOX) [1]. Arachidonic acid metabolism by 5-LOX requires the 5-LOX-activating protein (FLAP) for the formation of two groups of leukotrienes: the dihydroxy acid leukotriene B_4_ (LTB_4_) formed by the action of LTA_4_ hydrolase (LTA_4_H); and the cysteinyl-leukotrienes (CysLTs—LTC_4_, D_4_, and E_4_) by that of LTC_4_ synthase. Each group acts on its own specific receptors (BLT and CysLT receptors, respectively) [2]. In addition to their roles in innate immune responses, LTs are implicated in cerebrovascular diseases with emerging evidence pointing to their involvement in atherosclerotic processes in the cardio-cerebral vasculature [3, 4]. Pathological studies on human atheromatous plaques and atherosclerotic coronary arteries revealed increased expressions of 5-LOX, FLAP, LTA_4_H, and leukotriene C_4_ [5–7].

LTB_4_ has been shown to activate and recruit leukocytes [8] and T cells [9], thereafter stimulating the production of cytokines and nuclear factor-kappaB (NF-κB) [10]. In animal studies, cerebral ischemia has been shown to activate 5-LOX and upregulate its expression, whereas 5-LOX inhibition reportedly confers neuroprotective effects against ischemic brain injuries [11–13]. Zileuton, a 5-LOX inhibitor that is in clinical use as an anti-asthmatic drug, has been found to reduce edema, inflammation, neuronal apoptosis, and infarct volume in experimental stroke models [14–16]. However, these previous studies did not evaluate and relate the observed protective effects of zileuton to the reduction of any particular 5-LOX product. Under ischemic conditions following a stroke, tissue glutathione (GSH) is rapidly consumed and depleted [17]. As GSH is required for the synthesis of LTC_4_ from LTA_4_, production of CysLTs might diminish or stop during cerebral ischemia, potentially leading to a selective overproduction of LTB_4_. Therefore, one might expect that LTB_4_ may play a predominant role in stroke pathology when compared to other LTs. However, the effects of LTB_4_ or LTB_4_ antagonism in brain ischemic injuries remain uninvestigated.

In human studies, Katsura et al. [18] were the first to report elevated levels of plasma LTs in patients with cerebrovascular diseases, suggesting involvement of LTs in stroke pathology. In post-mortem studies, some neuronal 5-LOX upregulation was observed in the cerebral cortex from a hypoxemic patient, and 5-LOX-immunopositive glial cells appeared in the foci of ischemic damage [19]. Genetic association studies indicate that the leukotriene pathway confers increased risk for myocardial infarction and ischemic stroke. Bevan et al. [20] found that a haplotype in the LTB_4_ receptor complex confers a 2.3-fold increase in risk of cardioembolic stroke. Helgadottir et al. [21] from the DeCODE group identified a 4-marker single nuclear polymorphism (SNP) termed Hap A in the arachidonate 5-lipoxygenase-activating protein (ALOX5AP) gene locus that confers a 1.67 greater risk of myocardial infarction and stroke in an Icelandic population. However, another study found no association of two polymorphisms (rs10507391 and rs12429692) in the ALOX5AP with ischemic stroke risk in a Han Chinese population [22]. In the latter study, plasma LTB_4_ levels were reported to be significantly higher in ischemic stroke cases than in controls, and carriers of the T allele of the rs10507391 variant were associated with higher plasma LTB_4_. To date, elevated plasma LTB_4_ levels in ischemic stroke patients had not been linked to either stroke severity or clinical outcome. With this background, we embarked on a study to investigate the relationship of LTB_4_ levels with stroke severity and clinical outcome in a prospective cohort of ischemic stroke patients who suffered middle cerebral artery infarction. Based on our observations in the clinical studies, we then proceeded to substantiate our human data by animal studies using experimental stroke models.

## Methods

### Patients

A total of 25 patients who were diagnosed with acute middle cerebral artery infarction and presented < 4.5 h from stroke onset at the National University Hospital, Singapore, were recruited for this study. Risk factors, including hypertension, hyperlipidemia, diabetes mellitus, cigarette smoking, and atrial fibrillation, were collected and verified against medical records. Patients < 21 years old with pregnancy, intracranial hemorrhage, active cancer, and autoimmune diseases were excluded. Stroke severity was assessed according to the National Institute of Health Stroke Scale (NIHSS). Healthy controls, comprising age-matched individuals without stroke, myocardial infarction, cancer, and autoimmune diseases, were recruited. After obtaining informed consent, whole blood was collected from each patient in EDTA tubes, before administration of recombinant tissue plasminogen activator (rtPA) (day 0). Blood collection was repeated on days 1 and 7. Mean collection time on day 0 was 150 (SD = 20) min after stroke onset. Whole blood was centrifuged at 2800 rpm, 4 °C for 10 min, and preserved with 15 μM indomethacin and 40 μM butylated hydroxytoluene, to prevent oxidation during storage at − 80 °C. Functional outcomes were determined on day 90 post-stroke using the modified Rankin scale (MRS) where MRS 0–2 was considered as good outcome and MRS 3–6 as poor outcome. The study protocol was approved by the Domain-Specific Review Board, National Healthcare Group, Singapore, and all subjects provided written informed consent prior to their study participation.

### Measurements of Neutrophil and Leukotriene B_4_

Neutrophil counts were measured using the Sysmex pocH-100i automated hematology analyzer (Sysmex Corp, Kobe, Japan). Plasma and tissue levels of LTB_4_ were measured by gas chromatography mass spectrometry (GCMS) [23]. Briefly, LTB_4_-d4 (20 ng) was added to each sample as an internal standard. Solid-phase extraction was performed by using Agilent Bond Elute Certify II cartridges. Sodium formate (100 mM, 1 mL) was added into the sample and vortexed briefly. The pH was adjusted to 3.0 with formic acid (2 M, 20 μL). Samples were eluted through the conditioned column and washed with water (1 mL) followed by elution with ethyl acetate (1 mL). Ethyl acetate was completely evaporated under ultra-high purity nitrogen gas (99.9%). Samples were then incubated with 15 μL DIPEA (10%, v/v, acetonitrile) and 30 μL PFBBr (10%, v/v, acetonitrile) at room temperature for 30 min and dried under nitrogen gas. To the dried samples, 20 μL of acetonitrile and 40 μL of BSTFA with 1% TMCS were added and incubated at 40 °C for 1 h for silylation of the LTB_4_. The samples were subsequently dried and reconstituted in 35 μL isooctane. Samples were injected into an Agilent 7890A GC coupled with 5975 °C mass spectrometer. The stationary phase used was Agilent DB1701 capillary column 30 m, 0.25 μm diameter. The mobile phase was helium at a flow rate of 1 mL/min in a negative chemical ionization mode. LTB_4_ was quantified by monitoring the M-PFB ion at m/z 479 for LTB_4_ and corresponding ion at m/z 483 for the LTB_4_-d4 using the selected ion monitoring (SIM) scan mode. Triplicate measurements were done for each sample.

### Embolic Stroke Model

All animal care and experimental procedures in this study were approved by the Institutional Animal Care and Use Committee of the National University of Singapore and complied with NIH guidelines for the care and use of laboratory animals. Animals were housed under diurnal lighting conditions and fed standard rat chow and water ad libitum. Male Wistar rats (6–8 weeks old) were anesthetized with isoflurane (1.5%) in a 30% oxygen and 70% nitrous oxide mixture. Body temperature was maintained at 37 °C until recovery from anesthesia. The embolic stroke model was adapted from Zhang et al. [24] and Aoki et al. [25]. Briefly, femoral arterial blood from a donor rat was withdrawn into a PE-50 tube and kept at room temperature for 2 h. PE-50 tube containing the extracted blood was transferred to 4 °C for 22 h to form a clot. The clot was subsequently cut into 3.5-cm sections and transferred into sterile saline. After several washes, the clots were gently shifted to a modified PE-50 catheter.

A surgical incision was performed via the ventral surface of the neck to expose the internal and external carotid arteries. The external carotid artery and pterygopalatine artery were permanently ligated, while the occipital artery was cauterized. Surgical clips were applied to the common and the internal carotid arteries to stop blood flow during incision on the external carotid artery. The modified PE-50 catheter containing the blood clot was inserted gently through the external carotid artery into the distal internal carotid artery to occlude the MCA. The catheter was withdrawn 5 min after the clot was injected. Laser Doppler Flowmetry (LDF) (2 mm posterior, 5 mm lateral to bregma) was used to monitor cerebral blood flow. A relative reduction in cerebral blood flow of > 70% from pre-ischemic baseline following occlusion being maintained for > 2 h without spontaneous recanalization was used as a pre-requisite in this stroke model. Total number of animals used was 49 with a mortality rate of approx. 20%.

At 3 or 24 h after the embolic stroke induction, the rats were euthanized and whole blood was collected and then centrifuged at 2800 rpm, 4 °C for 10 min, and preserved with 15 μM indomethacin and 40 μM butylated hydroxytoluene, to prevent oxidation during storage at − 80 °C. Rats were perfused through the heart with phosphate buffered saline (PBS) and the brains were harvested. Cortex from ipsilateral and contralateral side of the brains were isolated and kept frozen at − 80 °C until use.

### Transient Middle Cerebral Artery Occlusion Model

Animals used were as described for the ES model. Transient middle cerebral artery occlusion (tMCAO) model was achieved following the surgical procedure described above except that a silicon-coated 4.0 nylon monofilament was inserted to occlude the MCA as described previously [25, 26]. Briefly, a silicon-coated 7–0 polypropylene monofilament was inserted into the external carotid artery and advanced to the internal carotid artery until a significant fall in cerebral blood flow to around 30% of baseline by LDF. The filament was withdrawn after 60 or 100 min of occlusion. Total number of animals used was 36 with mortality rates of approx.10% and 40%, respectively, for 60 and 100 min tMCAO.

### Drug Administration

LTB_4_ (50 ng, Cayman USA) was dissolved in 0.5 μL ethanol and diluted to 1 mL using sterile PBS, and then infused at a rate of 2 mL/h (KD Scientific syringe pump) through a polyethylene catheter (PE-10) into the internal carotid artery immediately prior to tMCAO induction. Vehicle-treated controls were similarly infused with PBS containing 0.05% ethanol. BAY-X1005 (0.2 mg/kg, Tocris Biosciences USA) and LY255283 (1 mg/kg, Tocris Biosciences USA) dissolved in 1% DMSO in sterile saline were administered by intraperitoneal injection (1 mL/kg) 10 min before tMCAO.

### Measurement of Infarct Volume and Immunofluorescence Studies

Twenty-four hours after the induction of tMCAO, rats were anesthetized and perfused with 0.1 M PBS followed by 4% paraformaldehyde through the heart. Brains were removed and post-fixed in 4% paraformaldehyde overnight at 4 °C and then cryoprotected in 10% and then 20% sucrose at 4 °C until use. Coronal sections (30 μm) were made using a cryostat (Leica 3050S, Germany), mounted onto glass slides (Matsunami, Japan) stained with 0.25% thionin (Sigma, USA) and then dehydrated through increasing concentration of ethanol. Infarction volumes were quantified on thionin-stained section using ImageJ (NIH, USA) in a blinded manner and presented as percentage of the total brain volume. For immunofluorescent staining, non-specific binding was blocked by incubating the section in 5% goat serum for 1 h at room temperature. Antibodies against 5-LOX (1:4000, BD Bioscience, Cat. No. 610694), LTA_4_-hydrolase (1:400, Abcam Cat. No. ab133512) or MPO (1:200, Novus Biologicals, Cat. No. AF3667) were used and incubated at 4 °C overnight. Sections were then rinsed and incubated with Alexa 488-conjugated goat anti-rabbit or Alexa 555-conjugated goat anti-mouse (Molecular Probes, USA) secondary antibody for 1 h at room temperature. Fluorescent-labeled sections were covered using Prolong-Gold antifade mounting medium (Life Technologies, USA). Negative control was performed by probing sections with PBS and 0.1% Triton-X overnight. All fluorescent images were captured using an Olympus FluoViewFV1000 (Olympus, Japan) laser scanning confocal microscope and all images were processed by FV10-ASW1.7.

### Western Blotting

The cortex was dissected from control and embolic stroke rats according to Spijker [27]. Cortical tissues were lysed by RIPA buffer (Cell Signaling Technologies, USA) supplemented with a protease inhibitor cocktail (Roche, Mannheim, Germany). Total protein was determined by the Bradford protein assay (Bio-Rad, CA, USA). Proteins were separated by 10% SDS/PAGE, transferred onto a PVDF membrane (Amersham Biosciences, UK) and then blocked with 10% non-fat milk. The membrane was then incubated with antibodies against 5-LOX (1:1000, BD Bioscience), GFAP (1:5000, Chemicon, Merck, USA, Cat. No. MAB3402), ED-1 (1:1000, Chemicon, Merck, Germany, Cat. No. MAB1435), OX-42 (1:1000, Chemicon, Merck, Germany, Cat. No. CBL1512), or β-actin (Cell Signaling Technologies, USA, Cat. No. mAb#4970) at 4 °C overnight, then washed and incubated in HRP-conjugated anti-rabbit or mouse IgG at room temperature for 1 h. Visualization was carried out using Luminata Forte or Crescendo Western HRP substrate (Millipore Corporation, USA) and chemiluminescence signals were detected using UVIchemi (UVItec, UK).

### Statistical Analysis

Statistical analyses were performed using the IBM SPSS software (Version 22, SPSS Inc., USA). In human studies, data analyses were performed using Wilcoxon signed ranks tests for related variables and Mann-Whitney *U* tests for unrelated variables. Friedman test for related variables with correction for multiple comparisons was performed to examine temporal relationship of plasma LTB_4_ levels in stroke patients. Adjustments for potential confounders were made using logical regression methods. Power calculations, performed a priori based on our pilot data, indicated that at least 24 subjects would be required to detect a 20% change in plasma LTB_4_ from day 0 to day 1 with 80% power using 2-tailed *t* test with statistical significance set at *p <* 0.05. In animal studies, data analyses were performed using one-way or two-way ANOVA followed by post hoc analysis as indicated in the figure legends, or by independent Student’s *t* test.

## Results

### Temporal Release of LTB_4_ in Patients with Acute Middle Cerebral Artery Infarction

This study was designed to investigate the plasma LTB_4_ levels in ischemic stroke patients on days 0, 1, and 7 post-stroke, and whether plasma LTB_4_ levels relate to the initial stroke severity or 90-day functional outcome. A total of 25 subjects (mean age, 62 years; 64% men) diagnosed with acute ischemic stroke by computed tomography or magnetic resonance imaging studies were recruited. Their median presenting National Institute of Health Stroke Scale (NIHSS) was 19 (interquartile range, 14–24); 76% of patients had hypertension, 40% diabetes mellitus, 16% atrial fibrillation, and 56% dyslipidemia prior to their stroke presentation (Table 1). All patients received intravenous rtPA treatment (0.9 mg/kg) and completed the 90-day follow-up.

**Table 1:** Clinical characteristics of stroke patients and controls

	Stroke patients (*n* = 25)	Controls (*n* = 16)	*p* values
Demographics			
Age (years)	62 (55–72)	58 (54–68)	0.846
Gender (%)			0.342
Men	64	56	
Women	36	44	
Risk factors (%)			< 0.001
Hypertension	76	13	
Hyperlipidemia	56	6	
Diabetes mellitus	40	6	
Cigarette smoking (ever or current)	12	0	
Atrial fibrillation	16	0	

Age is expressed as median (interquartile range). *p* values obtained by Mann-Whitney *U* test for continuous variables and chi-squared test for categorical variables

**Fig. 1:**
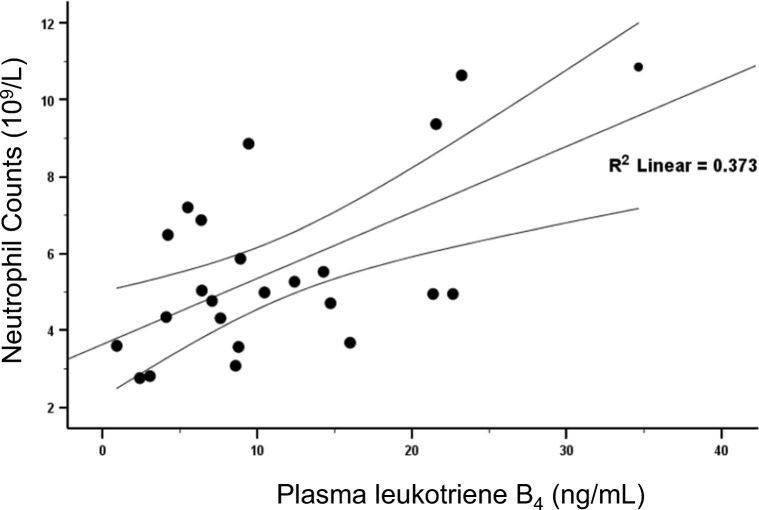
Correlation of neutrophil counts with LTB_4_ levels in the plasma of ischemic stroke patients on day 0. Significant correlation was obtained by Spearman rank-order correlation, *r*^2^ = 0.373, *p* = 0.018, *n* = 25. Graph shows linear regression line with 95% confidence limits

**Fig. 2:**
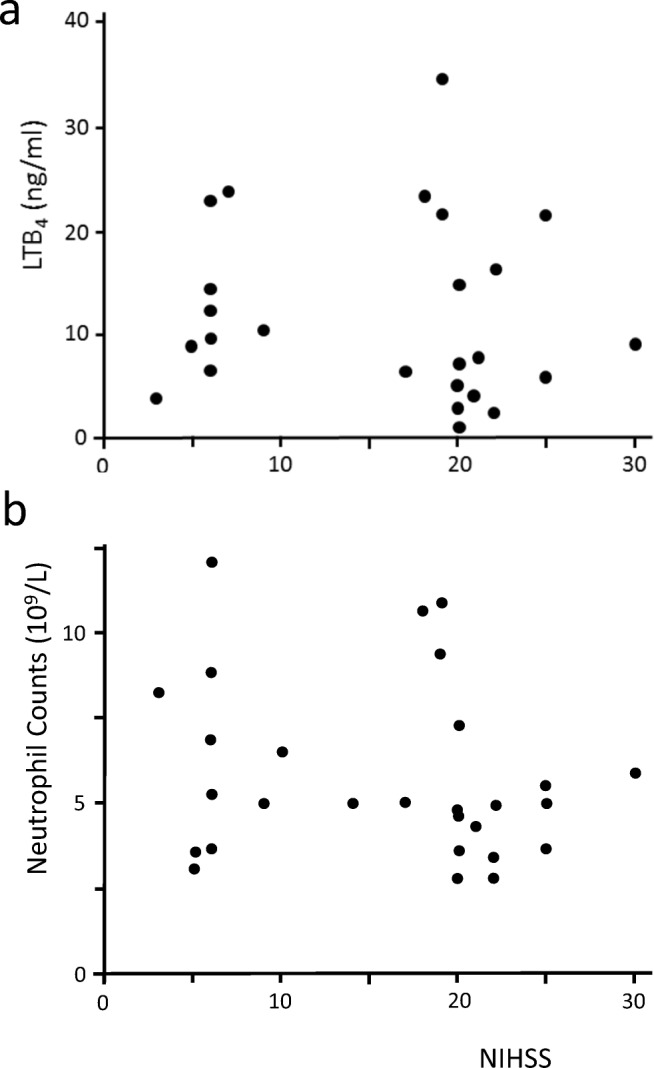
Correlation of stroke severity with (**a**) LTB_4_ levels and **(b**) neutrophil counts in the plasma of ischemic stroke patients on day 0. No significant correlations were obtained by Spearman rank-order correlation, *r*^2^ = 0.002, *p* = 0.955 (**a**) and *r*^2^ = 0.054, *p* = 0.236 (**b**), *n* = 25

**Fig. 3:**
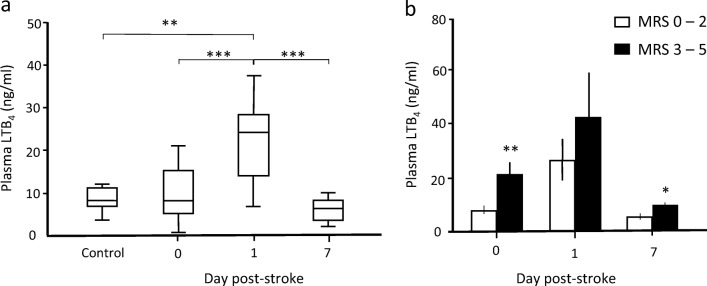
Correlation between plasma LTB_4_ levels in stroke patients with clinical outcome following acute middle cerebral artery infarction. (**a**) LTB_4_ levels in plasma of healthy controls (*n* = 16) and stroke patients on days 0, 1, and 7 (*n* = 25). **Significant difference between control and day 1 by Mann-Whitney *U* test *p* = 0.03. ***Significant difference between day 0 (baseline) and day 1, and between day 1 and day 7 stroke samples by Friedman test for related variables with correction for multiple comparisons, *p* < 0.01. (**b**) Comparison of plasma LTB_4_ levels on days 0, 1, and 7 between patients stratified by good (MRS 0–2, *n* = 15) vs poor (MRS 3–5, *n* = 10) clinical outcome as assessed by functional recovery on day 90 post-stroke. *p < 0.05, **p < 0.01 between good and poor clinical outcomes adjusted for age and stroke severity by logistics regression methods

When neutrophil counts obtained on day 0 were plotted against LTB_4_ levels (Fig. 1), a statistically significant correlation was demonstrated (*r*^2^ = 0.373, *p* = 0.018). In contrast, neither neutrophil counts nor LTB_4_ levels correlate with the NIHSS score on day 0 (Fig. 2), demonstrating that these two parameters have no detectable influence on the initial stroke severity. Figure 3a shows that plasma LTB_4_ levels in healthy controls were comparable with baseline stroke levels, while a significant 2-fold increase in plasma LTB_4_ levels was observed from day 0 to day 1 post-stroke. By day 7, plasma LTB_4_ levels were lower than those on day 0. No significant differences in plasma LTB_4_ levels were observed between gender groups in healthy controls and stroke patients (Table 2). Ninety days following stroke onset, 15 (60%) patients had a good functional outcome (MRS 0–2) and 10 (40%) patients had a poor outcome (MRS 3–5). When stratified by clinical outcomes at 90 days, patients with a poor functional recovery had significantly higher plasma LTB_4_ levels on days 0 and 7 (*p* < 0.01, Mann-Whitney *U* test); the difference observed on day 1 did not reach statistical significance (*p* = 0.07, Mann-Whitney *U* test) (Fig. 3b). We identified age and stroke severity as two potential confounding factors. After correction, our findings remained significant.

**Table 2:** Comparison of plasma LTB_4_ levels in males and females

	Male	Female	*p* values
Control	8.5 (7.3–16.4)	11.8 (6.5–15.2)	0.685
Patients (day 0)	9.1 (6.0–15.1)	8.9 (4.2–21.5)	0.959
Patients (day 1)	24.6 (10.6–28.4)	22.9 (8.7–53.8)	0.920
Patients (day 7)	6.1 (2.3–8.0)	8.0 (5.7–9.3)	0.421

Plasma LTB_4_ as expressed as median (interquartile range) in ng/mL. *p* values obtained by Mann-Whitney *U* test

### LTB_4_ Levels in the Ipsilateral Cortex and Plasma After Embolic Stroke in Rats

**Fig. 4:**
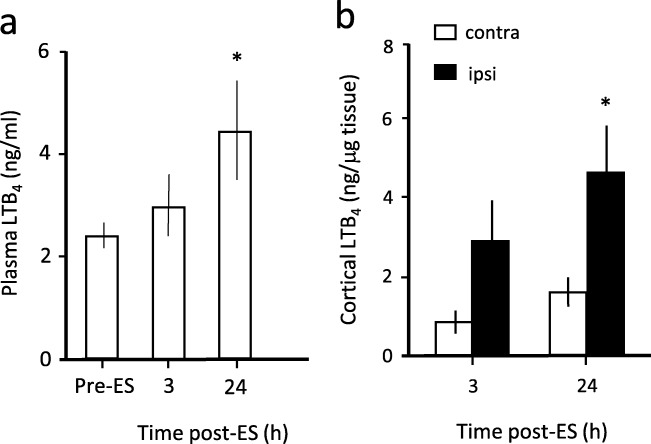
LTB_4_ levels in plasma and cortex of thromboembolic stroke (ES) rats 24 h post-stroke. (**a**) Plasma LTB_4_ levels for pre-ES control (*n* = 24), and at 3 (*n* = 15) and 24 h (*n* = 9) post-ES. ***p < 0.001 against pre-ES or 3 h post-ES by one-way ANOVA followed by Tukey post hoc test. (**b**) LTB_4_ levels in the ipsilateral (ipsi) and contralateral (contra) cortex after ES (*n* = 4). * *t =* 2.581, *p* < 0.05 against the contralateral side by two-tailed independent *t* test. At 3 h, *t* = 2.029, *p* = 0.089. Total number of animals used was 24

Figure 4a shows that plasma LTB_4_ level was increased at 24 h but not at 3 h post-embolic stroke when compared to pre-stroke levels. Importantly, concomitant increase in LTB_4_ levels in the ipsilateral cortex of ES rats was observed (Fig. 4b), reaching statistical significance at 24 h but not at 3 h when compared to the contralateral side.

### Upregulation of 5-LOX and LTA_4_ Hydrolase Expressions in the Infarcted Cortex After Embolic Stroke in Rats

**Fig. 5:**
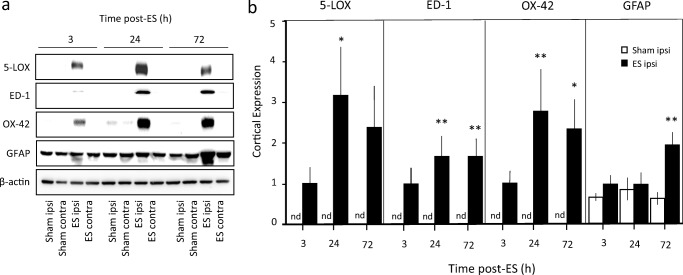
Expression of 5-LOX, ED-1, OX-42, and GFAP in the cerebral cortex after embolic stroke (ES). (**a**) Representative Western blots of 5-LOX, ED-1, OX-42, and GFAP in the ipsilateral (ipsi) and contralateral (contra) sides for sham and ES rats at 3, 24, and 72 h post-ES. (**b**) Comparison of cortical expression of 5-LOX, ED-1, OX-42, and GFAP between sham ipsi and ES ipsi, *n* = 3–5 per group. The ES group at 3 h was used as the reference group which was set to 1. Expressions of 5-LOX, ED-1, and OX-42 were consistently not detected (nd) in Sham controls. Statistical analysis was performed by two-way ANOVA testing time factor and stroke factor. *F*(1,14) = 13.83, *p* < 0.005 (5-LOX); *F*(1,24) = 35.65, *p* < 0.0001 (ED-1); *F*(1,18) = 21.64, *p* < 0.0005 (OX-42); *F*(1,14) = 14.14, *p* < 0.005 (GFAP), for stroke factor. *p < 0.05 and **p < 0.01 vs corresponding sham group by Bonferroni correction. No significance obtained for time factor and interaction in all 4 markers. Total number of animals used was 12

As shown in Fig. 5a, expression of 5-LOX, ED-1 (a marker for macrophages including phagocytic microglia), and OX-42 (a marker for microglia) was hardly detectable in either the sham-operated rats or on the contralateral side of the stroked rats. Increase in 5-LOX expression when compared to sham controls was observed in the cortex as early as 3 h after the ischemic insult but statistical significance was obtained only at 24 h (Fig. 5b). Parallel increases were observed in ED-1 and OX-42, also reaching statistical significance at 24 h and sustained until 72 h. On the other hand, GFAP (a marker for astrocytes) expression did not change within the first 24 h but significantly increased at 72 h, indicating that astrocyte activation/proliferation occur at a later time point than microglia activation.

**Fig. 6:**
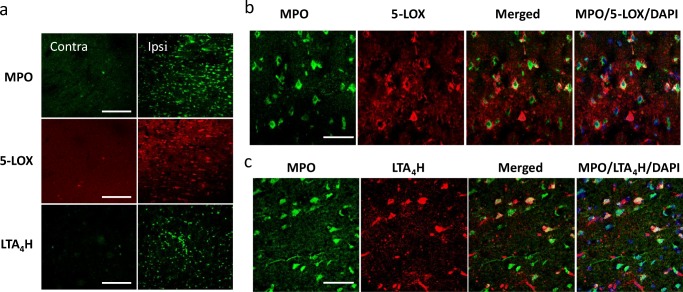
Immunofluorescence staining of MPO, 5-LOX, and LTA_4_H in the infarcted cortex 24 h post-ES. (**a**) Immunopositive cells increased many fold in the ipsilateral (ipsi) side when compared to the contralateral (contra) side showing marked upregulation of MPO, 5-LOX, and LTA_4_H expressions. Scale bar = 200 μm. (**b**) Double immunofluorescence staining showing 5-LOX and LTA_4_H are expressed in MPO-immunopositive neutrophils. Scale bar = 50 μm. Number of animals used was 4

Neutrophils are recruited to site of injury/inflammation within minutes through signals from chemoattractants including LTB_4_, which is the strongest lipid chemoattractant for neutrophils [28]. Using MPO as a neutrophil marker, the number of neutrophils was observed to be markedly increased in the infarcted ipsilateral cortex compared with the contralateral cortex, as previously observed [29]. Similarly, concomitant increases in the number of 5-LOX- and LTA_4_H-immunopostitive cells were observed (Fig. 6a). Both 5-LOX and LT_4_H immunoreactivity were observed in MPO-immunopositive cells, indicating that these two LTB_4_ synthesizing enzymes are expressed in neutrophils (Fig. 6b, c). However, it is apparent that not all MPO-immunopositive neutrophils express these two enzymes while there are also MPO-negative cells expressing either enzyme.

### Effects of LTB_4_, a FLAP Inhibitor and a BLTR Antagonist on Infarct Volume in tMCAO Rats

**Fig. 7:**
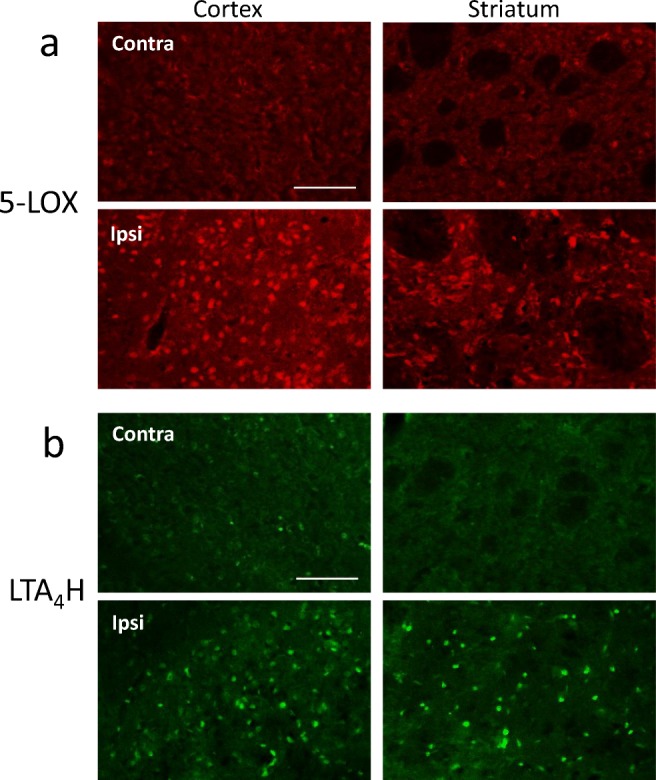
Upregulation of 5-LOX (**a**) and LTA_4_H (**b**) in the ipsilateral cortex and striatum 24 h after tMCAO (100 min). Scale bar = 100 μm. Total of animals used was 4

**Fig. 8:**
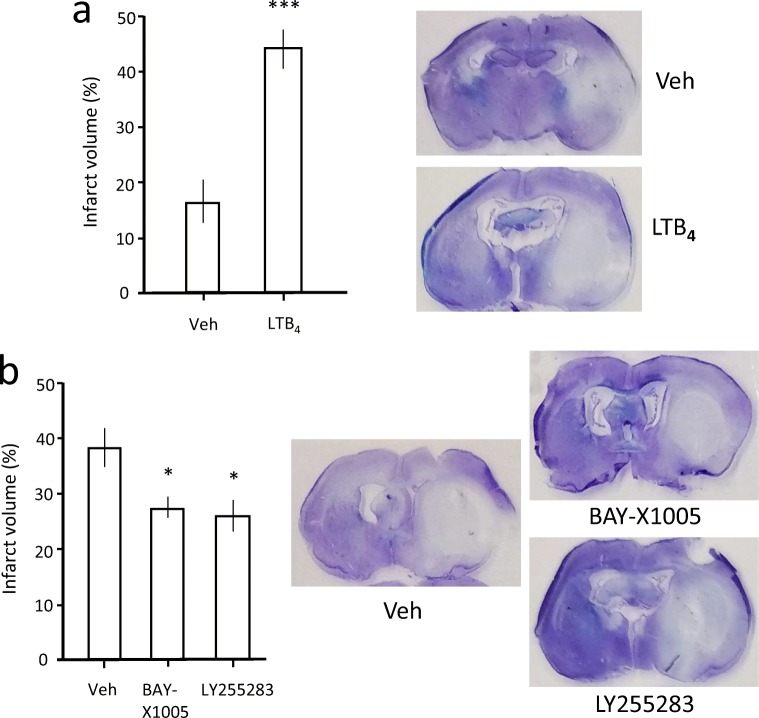
Effects of LTB_4_, a FLAP inhibitor (BAY-X1005) and a BLTR antagonist (LY255283) on infarction volume after tMCAO. (**a**) LTB_4_ loading by intracarotid infusion over 30 min before tMCAO (60 min) increased infarct volume, *n* = 4 per group. ***p < 0.001 against the vehicle group by two-tailed independent *t* test. (**b**) Administration of BAY-X1005 (0.2 mg/kg, i.p.) and LY255238 (1 mg/kg, i.p.) 10 min prior to tMCAO (100 min) reduced infarct volume, *n* = 4 per group. *p < 0.05 against the vehicle group by one-way ANOVA followed by Tukey post hoc test. Representative thionin-stained sections for each treatment group are presented. Total number of animals used was 20

To investigate further the effects of LTB_4_ on stroke outcome, we employed the tMCAO model instead of the thromboembolic model as it provides easier manipulation on the severity of ischemic insult simply by adjusting the duration of occlusion. Similar to the ES model, the upregulations of 5-LOX and LTA_4_H expression were confirmed in this model as shown in Fig. 7. LTB_4_ loading, via intracarotid infusion over 30 min before tMCAO (60 min), increased infarct volume by more than 2-fold at 24 h post-tMCAO (Fig. 8a) when compared to the vehicle-treated group. By contrast, the FLAP inhibitor BAY-X1005, which reduces LTB_4_ synthesis, and the BLTR antagonist LY255283 injected intraperitoneally 10 min prior to tMCAO (90 min) decreased the infarct volume by about 30% (Fig. 8b), corroborating previous findings that zileuton reduced infarct volume and improved neurological outcomes in a murine model of transient and global brain ischemia [15]. Taken together, these results strongly indicate that an increase in LTB_4_ production during the acute stages of ischemic stroke is detrimental and can worsen the extent of tissue infarct.

## Discussion

In the present prospective cohort study of ischemic stroke patients, we profiled LTB_4_ levels during the early stages of ischemic stroke and observed striking similarities in the temporal release of circulatory LTB_4_ in plasma of stroke patients, peaking at 24 h from symptom onset to almost twice the initial post-stroke levels, consistent with previous observation. While baseline LTB_4_ levels do not appear to relate to initial stroke severity, higher LTB_4_ levels on days 0 and 7 are associated with poor 90-day functional recovery (MRS 3–5). The peak LTB_4_ levels observed on day 1 did not differ between the good and poor outcome groups. These data indicated that poor functional recovery is associated with earlier and more sustained increase in LTB_4_ rather than the magnitude of the peak levels. As all stroke patients in this study received intravenous rtPA, which has been reported to decrease neutrophil activation [30], there is a possibility that the extent of the LTB_4_ increase might have been even higher if rtPA had not been used in these patients. However, as all patients received the same standard rtPA treatment, the data remained intrinsically comparable and thus the use of rtPA is unlikely to be material to our interpretation of the results. However, as only patients with hemispheric middle cerebral artery infarction were included, it remains unclear whether these findings can be extrapolated to patients with milder non-hemispheric strokes.

As shown in the ES stroke model, the brain responds to the ischemic insult with acute and prolonged inflammatory processes which are characterized by activation of resident microglia and infiltration of various types of immune cells including macrophages and neutrophils [31]. In this study, we observed marked increase in the expression of 5-LOX in the ischemic cortex which coincided with tissue inflammation as evidenced by concomitant increases in markers for macrophages (ED-1) and microglia (OX-42) within the infarcted cortex. These observations are consistent with the well-established occurrence of microglia activation and macrophage infiltration following stroke [32]. In contrast, gliosis did not occur as rapidly as the increases in macrophages and microglia, consistent with reported observations [33–35]. The fact that 5-LOX, ED-1, and OX-42 were hardly detectable in non-infarcted tissues indicated that all these three markers were upregulated early by 3 h. The Western blot results were supported by immunohistochemical studies which showed a distinctive increase in 5-LOX-immunopositive cells within the infarcted cortex. LTA_4_H-immunopositive and MPO-immunopositive cells were similarly increased. These observations are supportive of an increased production of LTB_4_ in ischemic brain tissues. Double immunostaining revealed that both 5-LOX and LTA_4_H were expressed in the MPO-immunopositive neutrophils. However, both enzymes are also expressed in other cell types, consistent with previously reported increase in 5-LOX expression in neurons, astrocytes, and macrophages following focal cerebral ischemia [11, 36].

Similar to the ES model, both 5-LOX and LTA_4_H expressions are significantly upregulated in the rat tMCAO model. These findings are consistent with those by Zhou et al. [11] who reported significant increase in 5-LOX 12–24 h after reperfusion, before returning to non-ischemic levels 3 to 7 days later, as well as an increase in LTB_4_ within the ischemic cortex. Our results, however, differ as we observed a significant and sustained increase in LTB_4_ at 24 h. Although such an increase in LTB_4_ was attributed mainly to increased 5-LOX and LTA_4_H activities in neurons and astrocytes, the intense rise and co-localization of LTB_4_ with neutrophils (in our study) point to a significant contribution from neutrophils, probably through an increase in leukocyte infiltration into the brain from ischemia-induced inflammation, secretion of chemokines, and disruption of the blood-brain barrier [32]. It is noteworthy that the rise in LTB_4_ was observed both in the ischemic cortex and in plasma, suggesting that circulatory LTB_4_ may be a reliable surrogate of brain levels, which may in part be explained by a breakdown in the blood-brain barrier following cerebral ischemia. This observation strengthens the reliability of using plasma LTB_4_ levels in human studies.

To demonstrate the effects of increased LTB_4_ on ischemic injury, LTB_4_ was administered (50 ng) by intracarotid injection prior to tMCAO, a significant increase in infarct volume was observed. This is the first direct demonstration of a deleterious effect of LTB_4_ during cerebral ischemia, which strongly supports the idea that high LTB_4_ levels in the acute phase of a stroke could worsen ischemic injuries. With regard to the question of whether the rise in LTB_4_ triggers stroke progression or results from the cerebral ischemic insult, our data appear to indicate the former as we observed in animal studies that either inhibiting LTB_4_ production by a FLAP inhibitor (BAY-X1005) or blockade of BLTR with an antagonist (LY255283) resulted in a significant reduction in infarct volume. These observations corroborate with previous findings that zileuton reduced infarct volume and improved neurological outcomes in a murine model of transient and global brain ischemia [15]. Taken together, these results strongly indicate that an increase in LTB_4_ production during the acute stages of ischemic stroke is detrimental and can worsen the extent of tissue infarct. The similar extent of infarct reduction by these two agents suggests that LTB_4_ may be the predominant player in ischemia among the 5-LOX products. Moreover, BLTR antagonism should be a superior approach over 5-LOX inhibition as the latter is less selective and deplete multiple products, some of which may be beneficial rather than deleterious to ischemic injuries. As a case in point, it has been reported that lipoxin A_4_, which is an anti-inflammatory product of 5-LOX, is neuroprotective against brain ischemia through inhibition of 5-LOX translocation and LT synthesis [37].

There are two known subtypes of BLTR, namely BLT_1_ and BLT_2_, which are G protein-coupled receptors (GPCR). LY255283, once described as BLT_2_ specific [38, 39], is now known to be a non-selective antagonist that inhibits both BLT_1_ (non-competitively) and BLT_2_ (competitively)-mediated Ca^++^ mobilization [40, 41]. Therefore, the observed protective effects of LY255283 may involve either or both subtypes. Current evidence suggests that LTB_4_ causes the release of inflammatory chemokines and cytokines via BLT_1_ which appears to be involved in many inflammatory diseases with accumulation of neutrophils at the lesion site [41]. In contrast, the effects of BLT_2_ are more controversial. It has been reported that BLT_2_ activation led to elevation of reactive oxygen species (ROS) that was followed by activation of NF-κB, and conversely BLT_2_ downregulation using antisense BLT_2_ oligonucleotides attenuated inflammation and airway hyper-responsiveness [39]. On the other hand, BLT_2_ was reported not to be involved in mediating neutrophilic inflammation and instead may play a protective role in allergic airway inflammation [40]. Interestingly, a more recent study demonstrated that both BLT_1_ and BLT_2_ were rapidly upregulated after intracerebral hemorrhage (ICH) but BLT_1_ was upregulated to a greater extent. Moreover, when BLT_1_ knockout were compared to wild-type mice, substantially reduced neutrophil infiltration was observed in the former with no significant change in hematoma volume [42]. Unfortunately, BLT_2_ knockout mice were not investigated. Taken together, it may be argued that the LTB_4_ effects following an ischemic insult may involve both receptor subtypes with perhaps a more significant input from BLT_1_. It should also be noted that LTB_4_ binds preferentially to BLT_1_ when compared to BLT_2_, with a 20-fold difference in affinity [38]. However, at pathologically high concentrations of LTB_4_ such as those in the ischemic brain, the BLT_2_-mediated effects might become more prominent.

Several limitations merit mention. First, the small sample size may subject our findings to type 1 errors. Second, only patients with MCA stroke were included in this study, the findings may not be extrapolated to all stroke patients. It remains unclear whether these findings can be reproduced in patients with non-hemispheric stroke, and with milder neurologic deficits. Third, it would have been ideal to have a patient group without rtPA treatment for comparison.

In conclusion, we have presented several novel findings in this report. In human studies, early and sustained increases in plasma LTB_4_ levels in ischemic stroke patients are associated with poor clinical outcome. However, LTB_4_ appeared unrelated to initial stroke severity. In experimental stroke models, we demonstrated that (1) plasma and brain LTB_4_ levels increased concomitantly post-stroke, (2) LTB_4_ loading increased infarct volume, and (3) attenuation of LTB_4_ production and BLTR antagonism decreased infarct volume. These findings support LTB_4_ as a biomarker for poor clinical outcome in acute stroke patients. Further studies, including clinical trials, to investigate if LTB_4_ may serve as a therapeutic target for acute stroke pharmacotherapy may be warranted.

## Electronic supplementary material

ESM 1 (PDF 498 kb)

## Abbreviations

5-LOX: 5-Lipoxygenase
ALOX5AP: Arachidonate 5-lipoxygenase-activating protein
BLTR: B-Leukotriene receptor
CysLT: Cysteinyl-leukotriene
ES: Thromboembolic stroke
FLAP: 5-LOX-activating protein
GCMS: Gas chromatography mass spectrometry
GFAP: Glial fibrillary acidic protein
GPCR: G protein-coupled receptors
GSH: Glutathione
LDF: Laser Doppler flowmetry
LTA_4_H: Leukotriene A_4_ hydrolase
LTB_4_: Leukotriene B_4_
MPO: Myeloperoxidase
MRS: Modified Rankin scale
NF-κB: Nuclear factor-kappaB
PBS: Phosphate buffered saline
rtPA: Recombinant tissue plasminogen activator
tMCAO: Transient middle cerebral artery occlusion
